# Methyl 3-[(3,5-dichloro­anilino)carbon­yl]propionate

**DOI:** 10.1107/S1600536810001455

**Published:** 2010-01-16

**Authors:** B. S. Saraswathi, B. Thimme Gowda, Sabine Foro, Hartmut Fuess

**Affiliations:** a Department of Chemistry, Mangalore University, Mangalagangotri 574 199, Mangalore, India; b Institute of Materials Science, Darmstadt University of Technology, Petersenstrasse 23, D-64287 Darmstadt, Germany

## Abstract

In the title compound, C_11_H_11_Cl_2_NO_3_, the amide O atom and the carbonyl O atom of the ester segment are *anti* to each other and *anti* to the H atoms of the adjacent –CH_2_ groups. In the crystal structure, mol­ecules are packed into centrosymmetric dimers through inter­molecular N—H⋯O hydrogen bonds. The dimers are linked into a layer structure extending parallel to (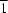
02) by C—H⋯O hydrogen bonds.

## Related literature

For related structures, see: Gowda *et al.* (2009**a* ▶,*b* ▶,c* ▶).
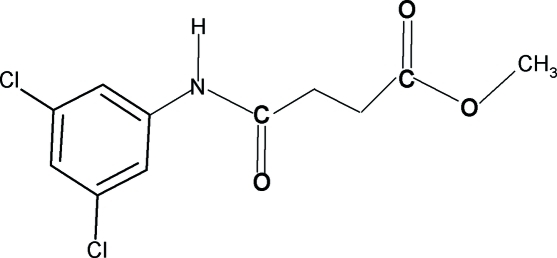


## Experimental

### 

#### Crystal data

C_11_H_11_Cl_2_NO_3_
*M* *_r_* = 276.11
Monoclinic, 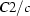

*a* = 12.865 (2) Å
*b* = 14.753 (3) Å
*c* = 14.114 (2) Å
β = 109.59 (2)°
*V* = 2523.7 (7) Å^3^
*Z* = 8
Mo *K*α radiation
μ = 0.51 mm^−1^
*T* = 299 K
0.50 × 0.16 × 0.12 mm

#### Data collection

Oxford Diffraction Xcalibur diffractometer with a Sapphire CCD detector
Absorption correction: multi-scan (*CrysAlis RED*; Oxford Diffraction, 2009 ▶) *T*_min_ = 0.785, *T*_max_ = 0.941
4503 measured reflections
2259 independent reflections
1453 reflections with *I* > 2σ(*I*)
*R*_int_ = 0.035

#### Refinement

*R*[*F* ^2^ > 2σ(*F* ^2^)] = 0.061
*wR*(*F* ^2^) = 0.166
*S* = 1.09
2259 reflections
157 parameters
1 restraint
H atoms treated by a mixture of independent and constrained refinement
Δρ_max_ = 0.37 e Å^−3^
Δρ_min_ = −0.21 e Å^−3^

### 

Data collection: *CrysAlis CCD* (Oxford Diffraction, 2009 ▶); cell refinement: *CrysAlis RED* (Oxford Diffraction, 2009 ▶); data reduction: *CrysAlis RED*; program(s) used to solve structure: *SHELXS97* (Sheldrick, 2008 ▶); program(s) used to refine structure: *SHELXL97* (Sheldrick, 2008 ▶); molecular graphics: *PLATON* (Spek, 2009 ▶); software used to prepare material for publication: *SHELXL97*.

## Supplementary Material

Crystal structure: contains datablocks I, global. DOI: 10.1107/S1600536810001455/ci5017sup1.cif

Structure factors: contains datablocks I. DOI: 10.1107/S1600536810001455/ci5017Isup2.hkl

Additional supplementary materials:  crystallographic information; 3D view; checkCIF report

## Figures and Tables

**Table 1:** Hydrogen-bond geometry (Å, °)

*D*—H⋯*A*	*D*—H	H⋯*A*	*D*⋯*A*	*D*—H⋯*A*
N1—H1*N*⋯O2^i^	0.85 (2)	2.17 (2)	3.017 (4)	172 (4)
C4—H4⋯O1^ii^	0.93	2.45	3.379 (5)	174

Symmetry codes: (i) 
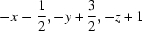
; (ii) 
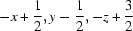
.

**Fig. 1.**
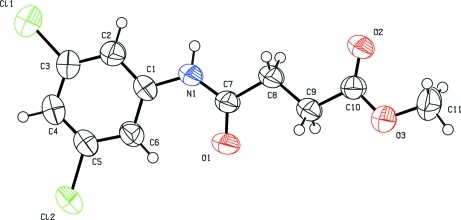
Molecular structure of the title compound, showing the atomic labelling scheme. Displacement ellipsoids are drawn at the 50% probability level. H atoms are represented as small spheres of arbitrary radii.

**Fig. 2.**
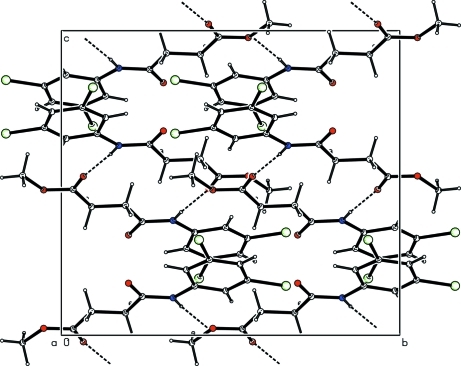
Part of the crystal structure of the title compound, showing hydrogen-bonded (dashed lines) dimers.

## supplementary crystallographic information

### Comment

As a part of studying the effect of ring and side chain substitutions on the structures of biologically significant compounds (Gowda *et al.*, 2009*a,b,c*), the crystal structure of *N*-(3,5-dichlorophenyl)methylsuccinamate [systematic name: 3-[(3,5-dichloro)-aminocarbonyl]propionate] has been determined.

The conformation of the amide O atom and the carbonyl O atom of the ester segment are *anti* to each other and both are *anti* to the H atoms of the adjacent -CH_2_ groups (Fig. 1), similar to that observed in *N*-(3,5-dichlorophenyl)methylsuccinamic acid (Gowda *et al.*, 2009*c*) and *N*-(3,5-dimethylphenyl)ethylsuccinamate (Gowda *et al.*, 2009*a*).

In the crystal, molecules are packed into centrosymmetric dimers through intermolecular N—H···O hydrogen bonds (Table 1 and Fig.2).

### Experimental

A solution of succinic anhydride (0.02 mol) in toluene (25 ml) was treated dropwise with a solution of 3,5-dichloroaniline (0.02 mol) in toluene (20 ml) with constant stirring. The resulting mixture was stirred for 1 h and set aside for an additional 1 hour at room temperature for the completion of reaction. The mixture was then treated with dilute hydrochloric acid to remove the unreacted 3,5-dichloroaniline. The resultant solid *N*-(3,5-dichlorophenyl)succinamic acid was filtered under suction and washed thoroughly with water to remove the unreacted succinic anhydride and succinic acid. It was recrystallized to constant melting point from methanol. Pure *N*-(3,5-dichlorophenyl)succinamic acid in methanol was refluxed with 2 ml of conc. sulfuric acid for 2 h and was subjected to slow evaporation. The resulting *N*-(3,5-dichlorophenyl)methylsuccinamate was recrystallized from methanol. The purity of the compound was checked and characterized by its IR and NMR spectra. Single crystals were grown in a methanol solution by slow evaporation at room temperature.

### Refinement

The H atom of the NH group was located in a difference map and refined with a N–H distance restraint of 0.86 (2) Å. The remaining H atoms were positioned geometrically [C–H = 0.93–0.97 Å] and refined using a riding model. All H atoms were refined with isotropic displacement parameters (set to 1.2 times of the *U*_eq_ of the parent atom).

### Crystal data

**Table:**

C_11_H_11_Cl_2_NO_3_	*F*(000) = 1136
*M**_r_* = 276.11	*D*_x_ = 1.453 Mg m^−^^3^
Monoclinic, *C*2/*c*	Mo *K*α radiation, λ = 0.71073 Å
Hall symbol: -C 2yc	Cell parameters from 1000 reflections
*a* = 12.865 (2) Å	θ = 3.0–28.1°
*b* = 14.753 (3) Å	µ = 0.51 mm^−^^1^
*c* = 14.114 (2) Å	*T* = 299 K
β = 109.59 (2)°	Needle, colourless
*V* = 2523.7 (7) Å^3^	0.50 × 0.16 × 0.12 mm
*Z* = 8	

### Data collection

**Table:**

Oxford Diffraction Xcalibur diffractometer with a Sapphire CCD detector	2259 independent reflections
Radiation source: fine-focus sealed tube	1453 reflections with *I* > 2σ(*I*)
graphite	*R*_int_ = 0.035
Rotation method data acquisition using ω and φ scans.	θ_max_ = 25.4°, θ_min_ = 3.0°
Absorption correction: multi-scan (*CrysAlis RED*; Oxford Diffraction, 2009)	*h* = −11→15
*T*_min_ = 0.785, *T*_max_ = 0.941	*k* = −17→10
4503 measured reflections	*l* = −17→16

### Refinement

**Table:**

Refinement on *F*^2^	Primary atom site location: structure-invariant direct methods
Least-squares matrix: full	Secondary atom site location: difference Fourier map
*R*[*F*^2^ > 2σ(*F*^2^)] = 0.061	Hydrogen site location: inferred from neighbouring sites
*wR*(*F*^2^) = 0.166	H atoms treated by a mixture of independent and constrained refinement
*S* = 1.09	*w* = 1/[σ^2^(*F*_o_^2^) + (0.0595*P*)^2^ + 4.6291*P*] where *P* = (*F*_o_^2^ + 2*F*_c_^2^)/3
2259 reflections	(Δ/σ)_max_ = 0.002
157 parameters	Δρ_max_ = 0.37 e Å^−^^3^
1 restraint	Δρ_min_ = −0.21 e Å^−^^3^

### Special details

**Table:**

Geometry. All e.s.d.'s (except the e.s.d. in the dihedral angle between two l.s. planes) are estimated using the full covariance matrix. The cell e.s.d.'s are taken into account individually in the estimation of e.s.d.'s in distances, angles and torsion angles; correlations between e.s.d.'s in cell parameters are only used when they are defined by crystal symmetry. An approximate (isotropic) treatment of cell e.s.d.'s is used for estimating e.s.d.'s involving l.s. planes.
Refinement. Refinement of *F*^2^ against ALL reflections. The weighted *R*-factor *wR* and goodness of fit *S* are based on *F*^2^, conventional *R*-factors *R* are based on *F*, with *F* set to zero for negative *F*^2^. The threshold expression of *F*^2^ > σ(*F*^2^) is used only for calculating *R*-factors(gt) *etc*. and is not relevant to the choice of reflections for refinement. *R*-factors based on *F*^2^ are statistically about twice as large as those based on *F*, and *R*- factors based on ALL data will be even larger.

### Fractional atomic coordinates and isotropic or equivalent isotropic displacement parameters (Å^2^)

**Table:**

	*x*	*y*	*z*	*U*_iso_*/*U*_eq_	
C1	0.0757 (3)	0.6059 (3)	0.6618 (3)	0.0532 (9)	
C2	0.0482 (3)	0.5146 (3)	0.6460 (3)	0.0591 (10)	
H2	−0.0243	0.4975	0.6116	0.071*	
C3	0.1280 (3)	0.4501 (3)	0.6814 (3)	0.0616 (11)	
C4	0.2370 (3)	0.4721 (3)	0.7337 (3)	0.0627 (11)	
H4	0.2906	0.4276	0.7580	0.075*	
C5	0.2620 (3)	0.5621 (3)	0.7481 (3)	0.0583 (10)	
C6	0.1848 (3)	0.6306 (3)	0.7149 (3)	0.0565 (10)	
H6	0.2048	0.6911	0.7275	0.068*	
C7	−0.0024 (3)	0.7610 (3)	0.6273 (3)	0.0560 (10)	
C8	−0.1087 (3)	0.8104 (3)	0.5736 (3)	0.0603 (11)	
H8A	−0.1369	0.7910	0.5040	0.072*	
H8B	−0.1632	0.7953	0.6047	0.072*	
C9	−0.0911 (3)	0.9108 (3)	0.5778 (3)	0.0624 (11)	
H9A	−0.0324	0.9245	0.5512	0.075*	
H9B	−0.0665	0.9298	0.6477	0.075*	
C10	−0.1908 (3)	0.9652 (3)	0.5209 (3)	0.0551 (10)	
C11	−0.2543 (4)	1.1146 (3)	0.4756 (4)	0.0849 (15)	
H11A	−0.3094	1.1103	0.5074	0.102*	
H11B	−0.2866	1.0998	0.4056	0.102*	
H11C	−0.2258	1.1753	0.4824	0.102*	
N1	−0.0090 (2)	0.6699 (2)	0.6230 (3)	0.0569 (9)	
H1N	−0.072 (2)	0.645 (3)	0.595 (3)	0.068*	
O1	0.0828 (2)	0.8016 (2)	0.6710 (3)	0.0875 (11)	
O2	−0.2818 (2)	0.93505 (18)	0.4784 (2)	0.0668 (8)	
O3	−0.1659 (2)	1.05231 (19)	0.5228 (2)	0.0754 (9)	
Cl1	0.09280 (11)	0.33629 (8)	0.66222 (11)	0.0926 (5)	
Cl2	0.39797 (8)	0.59326 (9)	0.81343 (10)	0.0845 (5)	

### Atomic displacement parameters (Å^2^)

**Table:**

	*U*^11^	*U*^22^	*U*^33^	*U*^12^	*U*^13^	*U*^23^
C1	0.0445 (19)	0.062 (2)	0.051 (2)	0.0079 (18)	0.0133 (17)	0.0045 (19)
C2	0.057 (2)	0.059 (3)	0.058 (2)	−0.0010 (19)	0.0150 (18)	−0.001 (2)
C3	0.067 (2)	0.056 (3)	0.064 (3)	0.010 (2)	0.024 (2)	0.004 (2)
C4	0.061 (2)	0.067 (3)	0.061 (3)	0.019 (2)	0.021 (2)	0.008 (2)
C5	0.048 (2)	0.070 (3)	0.056 (2)	0.0104 (19)	0.0149 (18)	0.002 (2)
C6	0.048 (2)	0.061 (2)	0.056 (2)	0.0050 (19)	0.0113 (18)	0.0050 (19)
C7	0.044 (2)	0.053 (2)	0.061 (3)	−0.0037 (18)	0.0048 (17)	0.0035 (19)
C8	0.045 (2)	0.057 (2)	0.066 (3)	−0.0019 (18)	0.0017 (18)	0.004 (2)
C9	0.045 (2)	0.058 (3)	0.072 (3)	−0.0013 (18)	0.0032 (19)	0.005 (2)
C10	0.052 (2)	0.054 (2)	0.055 (2)	−0.0041 (18)	0.0130 (18)	−0.0002 (19)
C11	0.096 (4)	0.058 (3)	0.101 (4)	0.018 (3)	0.033 (3)	0.010 (3)
N1	0.0402 (16)	0.055 (2)	0.065 (2)	−0.0013 (15)	0.0040 (15)	0.0031 (16)
O1	0.0455 (16)	0.0632 (19)	0.125 (3)	−0.0082 (14)	−0.0102 (16)	0.0064 (18)
O2	0.0458 (15)	0.0670 (18)	0.075 (2)	−0.0014 (13)	0.0035 (13)	0.0041 (15)
O3	0.0653 (18)	0.0543 (18)	0.095 (2)	0.0024 (14)	0.0120 (16)	0.0036 (16)
Cl1	0.0935 (9)	0.0592 (7)	0.1195 (12)	0.0048 (6)	0.0283 (8)	−0.0022 (7)
Cl2	0.0464 (6)	0.0885 (9)	0.1033 (10)	0.0137 (5)	0.0047 (6)	−0.0017 (7)

### Geometric parameters (Å, °)

**Table:**

C1—C2	1.391 (5)	C7—C8	1.510 (5)
C1—C6	1.401 (5)	C8—C9	1.496 (5)
C1—N1	1.407 (5)	C8—H8A	0.97
C2—C3	1.366 (5)	C8—H8B	0.97
C2—H2	0.93	C9—C10	1.499 (5)
C3—C4	1.387 (6)	C9—H9A	0.97
C3—Cl1	1.736 (4)	C9—H9B	0.97
C4—C5	1.365 (6)	C10—O2	1.207 (4)
C4—H4	0.93	C10—O3	1.322 (4)
C5—C6	1.384 (5)	C11—O3	1.440 (5)
C5—Cl2	1.743 (4)	C11—H11A	0.96
C6—H6	0.93	C11—H11B	0.96
C7—O1	1.219 (4)	C11—H11C	0.96
C7—N1	1.347 (5)	N1—H1N	0.850 (19)
			
C2—C1—C6	119.5 (4)	C7—C8—H8A	109.4
C2—C1—N1	117.8 (3)	C9—C8—H8B	109.4
C6—C1—N1	122.7 (4)	C7—C8—H8B	109.4
C3—C2—C1	119.8 (4)	H8A—C8—H8B	108.0
C3—C2—H2	120.1	C8—C9—C10	114.7 (3)
C1—C2—H2	120.1	C8—C9—H9A	108.6
C2—C3—C4	122.2 (4)	C10—C9—H9A	108.6
C2—C3—Cl1	119.4 (3)	C8—C9—H9B	108.6
C4—C3—Cl1	118.3 (3)	C10—C9—H9B	108.6
C5—C4—C3	116.9 (4)	H9A—C9—H9B	107.6
C5—C4—H4	121.5	O2—C10—O3	123.8 (4)
C3—C4—H4	121.5	O2—C10—C9	125.6 (4)
C4—C5—C6	123.6 (4)	O3—C10—C9	110.6 (3)
C4—C5—Cl2	118.7 (3)	O3—C11—H11A	109.5
C6—C5—Cl2	117.7 (3)	O3—C11—H11B	109.5
C5—C6—C1	117.9 (4)	H11A—C11—H11B	109.5
C5—C6—H6	121.1	O3—C11—H11C	109.5
C1—C6—H6	121.1	H11A—C11—H11C	109.5
O1—C7—N1	123.0 (4)	H11B—C11—H11C	109.5
O1—C7—C8	121.8 (4)	C7—N1—C1	128.6 (3)
N1—C7—C8	115.2 (3)	C7—N1—H1N	119 (3)
C9—C8—C7	111.0 (3)	C1—N1—H1N	113 (3)
C9—C8—H8A	109.4	C10—O3—C11	117.5 (3)
			
C6—C1—C2—C3	−0.8 (6)	O1—C7—C8—C9	3.2 (6)
N1—C1—C2—C3	179.3 (4)	N1—C7—C8—C9	−176.8 (4)
C1—C2—C3—C4	0.4 (6)	C7—C8—C9—C10	176.4 (4)
C1—C2—C3—Cl1	179.5 (3)	C8—C9—C10—O2	4.0 (6)
C2—C3—C4—C5	−0.6 (6)	C8—C9—C10—O3	−175.6 (4)
Cl1—C3—C4—C5	−179.6 (3)	O1—C7—N1—C1	−2.7 (7)
C3—C4—C5—C6	1.1 (6)	C8—C7—N1—C1	177.3 (4)
C3—C4—C5—Cl2	−180.0 (3)	C2—C1—N1—C7	−178.9 (4)
C4—C5—C6—C1	−1.5 (6)	C6—C1—N1—C7	1.2 (6)
Cl2—C5—C6—C1	179.6 (3)	O2—C10—O3—C11	3.3 (6)
C2—C1—C6—C5	1.3 (6)	C9—C10—O3—C11	−177.0 (4)
N1—C1—C6—C5	−178.8 (4)		

### Hydrogen-bond geometry (Å, °)

**Table:**

*D*—H···*A*	*D*—H	H···*A*	*D*···*A*	*D*—H···*A*
N1—H1N···O2^i^	0.85 (2)	2.17 (2)	3.017 (4)	172 (4)
C4—H4···O1^ii^	0.93	2.45	3.379 (5)	174

Symmetry codes: (i) −*x*−1/2, −*y*+3/2, −*z*+1; (ii) −*x*+1/2, *y*−1/2, −*z*+3/2.
